# Finding the Right Solvent: A Novel Screening Protocol for Identifying Environmentally Friendly and Cost-Effective Options for Benzenesulfonamide

**DOI:** 10.3390/molecules28135008

**Published:** 2023-06-26

**Authors:** Piotr Cysewski, Tomasz Jeliński, Maciej Przybyłek

**Affiliations:** Department of Physical Chemistry, Pharmacy Faculty, Collegium Medicum of Bydgoszcz, Nicolaus Copernicus University in Toruń, Kurpińskiego 5, 85-096 Bydgoszcz, Polandm.przybylek@cm.umk.pl (M.P.)

**Keywords:** benzenesulfonamide, solubility, deep learning, learning curve analysis, COSMO-RS, hyperparameters tuning

## Abstract

This study investigated the solubility of benzenesulfonamide (BSA) as a model compound using experimental and computational methods. New experimental solubility data were collected in the solvents DMSO, DMF, 4FM, and their binary mixtures with water. The predictive model was constructed based on the best-performing regression models trained on available experimental data, and their hyperparameters were optimized using a newly developed Python code. To evaluate the models, a novel scoring function was formulated, considering not only the accuracy but also the bias–variance tradeoff through a learning curve analysis. An ensemble approach was adopted by selecting the top-performing regression models for test and validation subsets. The obtained model accurately back-calculated the experimental data and was used to predict the solubility of BSA in 2067 potential solvents. The analysis of the entire solvent space focused on the identification of solvents with high solubility, a low environmental impact, and affordability, leading to a refined list of potential candidates that meet all three requirements. The proposed procedure has general applicability and can significantly improve the quality and speed of experimental solvent screening.

## 1. Introduction

In recent years, the importance of using green solvents has been emphasized to reduce the environmental impact of chemical processes [1,2,3,4]. Green solvents are those that are non-toxic and non-flammable and have a low environmental impact. Therefore, the use of green solvents is becoming increasingly popular in various fields, including pharmaceuticals [5], agrochemicals [6], and material science [7]. The development of a screening protocol for identifying environmentally friendly and cost-effective solvents for benzenesulfonamide is in line with this trend and can provide valuable insights for the development of greener chemical processes.

Solubility is a crucial property for both theoretical and practical applications of chemical compounds [8,9]. The ability to dissolve in a particular solvent can significantly impact the reactivity, stability, and bioavailability of a compound. In the pharmaceutical industry, solubility is a key factor in drug design and formulation [10,11,12,13]. Poor solubility can limit the efficacy and bioavailability of a drug, leading to increased costs and decreased patient compliance. Therefore, the development of efficient and effective methods for determining the solubility of compounds is of great importance [14,15,16,17].

Benzenesulfonamide (BSA) is a widely used compound in pharmaceuticals, agrochemicals, and material science [18,19,20,21,22]. Its characteristics have been the subject of numerous studies due to its importance as a building block for various applications [23,24,25,26]. Surprisingly, despite its importance, solubility has rarely been measured [27,28]. To the authors’ best knowledge, there is only one report presenting the solubility of BSA in neat solvents [28], and there are no data on its solubility in solvent mixtures. The most efficient solvents were found to be ketones, such as cyclopentanone, cyclohexanone, and acetone. Although these solvents are excellent for BSA dissolution, they can hardly be regarded as green ones. In general, cyclic ketones are commonly used as solvents in various industrial processes, including the production of pharmaceuticals, agrochemicals, and polymers [29,30,31,32]. Although cyclic ketones are generally not considered highly toxic solvents, they are recognized as VOC pollutants (volatile organic compounds), contributing to air contamination [33,34,35]. Additionally, these ketones can cause skin and eye irritation and may cause respiratory issues if inhaled in high concentrations [36]. In particular, exposure to cyclopentanone can cause headaches, dizziness, and nausea, and prolonged or repeated exposure can cause damage to the liver and kidneys [37,38]. Therefore, the development of a screening protocol for identifying environmentally friendly and cost-effective solvents for benzenesulfonamide would be a significant advancement in the field.

Extensive experimental screening, although the most reliable, is limited due to the time, effort, and costs needed. Hence, machine learning offers a real alternative for exploring the solvent space, provided that a reliable model has been developed [39,40,41,42]. As was established in previous studies [42,43], combining quantum chemical methods, such as COSMO-RS (Conductor-like Screening Model for Real Solvents) with machine learning methods, is a quite promising approach, providing good-quality predictions. However, in general, training models based on small datasets of features can pose several risks, including overfitting, a lack of diversity, limited accuracy, and limited applicability. One of the ways to overcome these potential shortcomings is the development of an ensemble of models [43,44,45,46]. Indeed, meta-models that incorporate a variety of base models can offer several advantages over a single model, especially if training is conducted on small datasets of molecular descriptors. By combining the predictions of multiple base models and meta-models, it is possible to achieve higher levels of accuracy, increased robustness to noise and outliers, better uncertainty estimation, and improved generalization to new, unseen data. This can be achieved because the base models may capture different aspects of the data, and by combining their predictions, the meta-model can provide a more comprehensive representation of the underlying patterns in the data. These benefits make meta-models a promising approach for a wide range of machine learning tasks, particularly those dealing with complex or high-dimensional datasets. The main goal of this paper is to present the effectiveness of such an approach for exploring the extended space of solvents to screen for new, efficient, environmentally friendly, and cost-effective solvents.

## 2. Results and Discussion

### 2.1. Solubility Measurements

**Figure 1:**
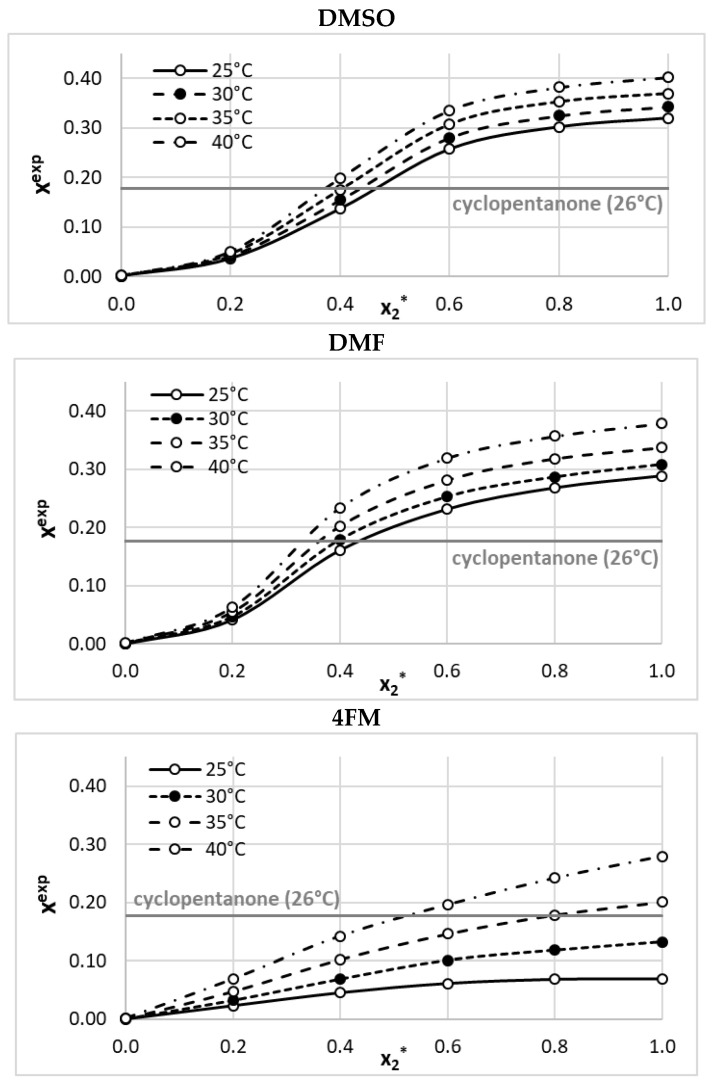
Mole fraction solubility of benzenesulfonamide in dimethylformamide (DMF), dimethyl sulfoxide (DMSO), and 4-formylmorpholine (4FM). On the abscissa, x_2_* denotes the mole fractions of a particular solvent in solute-free mixtures with water. Detailed results are available in Supplementary Materials (Table S1).

The experimentally determined BSA solubility–temperature profiles encompass neat and aqueous binary solvents, as presented in Figure 1 (tabularized data are provided in Supplementary Table S1). In all cases, a very high concentration of the solute was achieved. Notably, the aprotic polar solvents DMSO and DMF were found to be very effective solubilizers for sulfonamides, which was demonstrated in our previous studies [42,47]. This behavior is probably related to the effective solvation caused by the presence of hydrogen bonds, with the participation of the sulfonamide NH_2_ moiety playing the donating role, and lone electron pairs located on oxygen atoms in these solvents. This is why DMSO and DMF were found to exhibit significantly higher solubilizing abilities than cyclopentanone, which has been considered the most effective solvent so far.

Although, from the perspective of solvent screening, the solvation effects appear to be more crucial than the thermodynamic properties of the solid, the characteristics of the solid provide some interesting insights into solid–solution equilibria. In order to examine the potential effects of the solvents on the crystal form of the solute, the residues obtained after the shake-flask procedure were analyzed using DSC and FTIR-ATR methods. As can be inferred from Supplementary Figures S1 and S2, the formation of solvates or new polymorphs was not observed in any of the considered pure solvents (water, DMSO, DMF, and 4FM). Both spectra and thermograms recorded for the solid samples were similar to reference results obtained for pure chemicals. However, DSC measurements revealed some deviations from the melting point of the reference, especially in the case of DMF and 4FM. The onset values determined for the melting peaks in thermograms of the pure component, water, DMSO, DMF, and 4FM were 425.86, 426.64, 425.12, 423.15, and 424.43 K, respectively. These differences might be attributed to some impurities in the case of the residues obtained after the solubility determination procedure. This shows the inherent difficulties in drying compounds when utilizing the aforementioned solvents. On the other hand, low volatility is often regarded as a “green” feature [48,49], as compounds with lower vapor pressure tend to have a reduced potential for environmental mobility [50].

### 2.2. Ensemble Solubility Model

**Figure 2:**
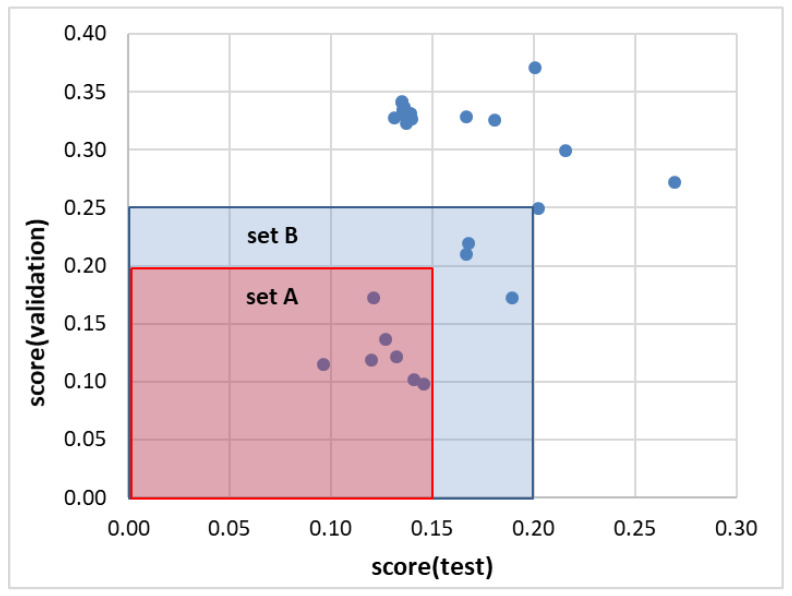
Results of regression model selection based on the distributions of loss values of test and validation sets. Set A comprises NuSVR, SVR, MLPRegressor, KNeighborsRegressor, GradientBoostingRegressor, CatBoostRegressor, and HistGradientBoostingRegressor. Set B includes AdaBoostRegressor, LGBMRegressor, and BaggingRegressor in addition to the previously selected regressors. Set A was defined by restricting it to regressors for which score values were less than 0.15 and 0.20 for test and validation subsets, respectively. Set B was augmented with regressors for which both criteria were relaxed by 0.05.

After training a set of regression models, the most representative ones were selected based on their performance on the test and validation subsets. It is good practice to tune the parameters of the models on the training set and verify their effectiveness using a portion of the data that have not been used before, which increases the predictability of the trained models. Figure 2 shows a scatter plot of the models’ characteristics, which enables the identification of two sets of regressors with similar efficiencies. To ensure the consistent prediction of solubility values, it is preferable to use a set of models instead of just a single one. The prediction of the ensemble defined by set A was compared to that of set B to ensure the consistency of the models’ performance on new cases. These results are provided in the Supplementary Materials (see models.xlsx and predictions.xlsx Excel files), along with the experimental data, all the descriptors used for model training, and the sets of tuned hyperparameters. Seven regressors, namely, NuSVR, SVR, MLPRegressor, KNeighborsRegressor, GradientBoostingRegressor, CatBoostRegressor, and HistGradientBoostingRegressor, were selected to form ensemble A and were used for predicting solubility values. Each of these regressors has its own strengths and weaknesses and may perform differently depending on the specific dataset and problem at hand. By combining their predictions, the ensemble can potentially reduce the variance and bias of the individual models and provide more robust predictions. While each of the regressors in the ensemble has its unique algorithm, there are some commonalities among them. For example, all of them are based on supervised learning, which means that they learn from labeled data to make predictions on new, unseen data. They are also based on the regression technique, which involves predicting a continuous output variable based on input features. Additionally, many of the regressors in the ensemble use ensemble learning techniques themselves, such as bagging and boosting, to improve their performance. All of this together suggests that the regressors in the ensemble are complementary and can work together to provide more accurate and reliable predictions of solubility values. The effectiveness of the ensemble in predicting solubility values was compared with another ensemble consisting of an extended set of regressors, as well as with each individual model’s performance.

It is not surprising that the neural network model, even with a simple multiple perceptron approach, achieved the highest accuracy on the test set. The model’s parameters, including the number of layers and neurons in each layer, were optimized using an Optuna study, resulting in a complex model with up to eight hidden layers with a varying number of neurons. However, such complexity can lead to overfitting, a common challenge in regression problems. To address this potential drawback, two methods were used in this paper, namely, a typical applicability domain plot (ADP) and a detailed learning curve analysis (LCA) from the scikit-learn 1.2.2 library [51]. While the former provides information on the distribution of standardized errors as a function of hat values, the latter is less commonly used but highly informative. Learning curve analysis is a valuable tool for evaluating the performance of machine learning models. It involves plotting the model’s training and validation performance as a function of the training set size, starting from a small sample and ending with the whole dataset. By analyzing the learning curves, one can extract several types of information, such as the model’s bias and variance, the optimal training set size, and the potential for further improvement. The bias and variance of the model can be estimated by examining the difference between the training and validation performance. The optimal training set size can be determined by observing the convergence of the learning curves. Finally, the slope of the learning curve can indicate the potential for further improvements in the model’s performance with more data. The application of LCA in this paper involved five-fold cross-validation to average test and training scores.

**Figure 3:**
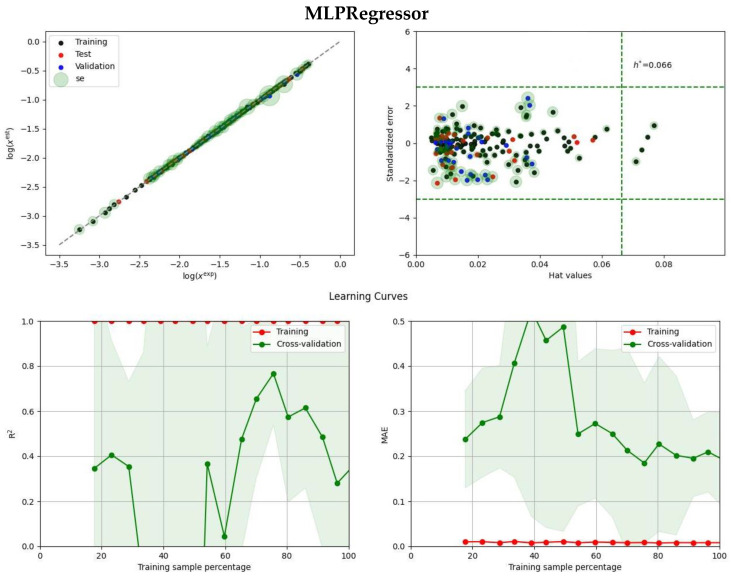

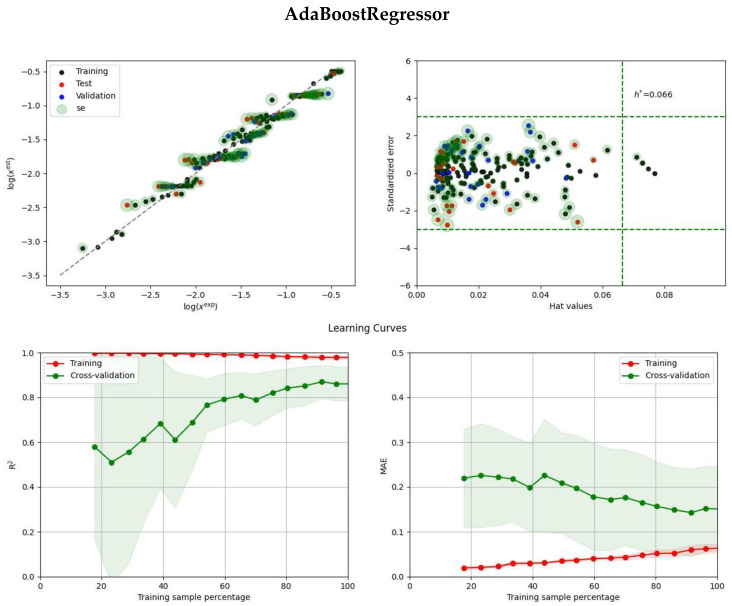
Illustration of the predictive power of MLPRegressor and AdaBoostRegressor. The rest of the models belonging to ensembles are depicted in Supporting Materials (see Table S2). The size of the green hollows represents the standard deviation values, while the green and red shadow regions on the learning curves represent the standard deviation of the training and cross-validation scores at each point. The dashed straight lines represent the h* value, which is the leverage threshold used to identify potential outliers in the dataset based on the hat values and standard error of the model in applicability domain analysis.

The Supplementary Materials contain comprehensive information on the models and the analyses performed (see Section S3). Furthermore, two regressors were subjected to detailed examination for illustrative purposes, as described below. Figure 3 presents an applicability domain plot and a learning curve analysis, both of which provide valuable insights. The MLPRegressor exhibits exceptional efficiency in training, even with a small fraction of the data, as evident from the almost-constant red line corresponding to either R^2^ (left panel) or MSE (right panel). The R^2^ value close to unity and the MAE value close to zero indicate the superior performance of this regressor on the training dataset. However, the green plots, which represent the test set performance, exhibit high sensitivity to the data subset included in the analysis. The absence of a definite trend in the green lines suggests overfitting. Despite the model’s high efficiency, its predictive potential may be considered problematic. Another regression model in ensemble A, the AdaBoostRegressor, exhibits distinct characteristics. As demonstrated in Figure 3, the accuracy of the training dataset is slightly dependent on the fraction of included data, resulting in a small reduction in R^2^ values and an increase in MAE values, which is anticipated behavior. The accuracy of the test subset is affected by the data fraction used in the analysis, but there is a visible saturation, as evidenced by the smooth and clearly trending green lines. These findings suggest that the predictive potential of this model is considerably higher and the obtained values for new cases are more reliable. Other regressors included in ensemble A share many commonalities, and they possess complementary properties, increasing the reliability of predictions made based on the entire ensemble. This expectation is further supported by the observation that extending the ensemble with additional models from set B does not alter the predicted values. This holds true not only for the data used in training and validation but also for the prediction of as many as 2067 new solvents used for screening purposes. It is worth mentioning that not all solvents used for predictions can be regarded for potential experimental validation since many were excluded due to not belonging to the applicability domain. However, such exclusions are not a weakness of the model but rather are related to the limited solvent space used for model training. The awareness of this fact is a crucial aspect of the reliability of model applications.

### 2.3. Screening of New Solvents

**Figure 4:**
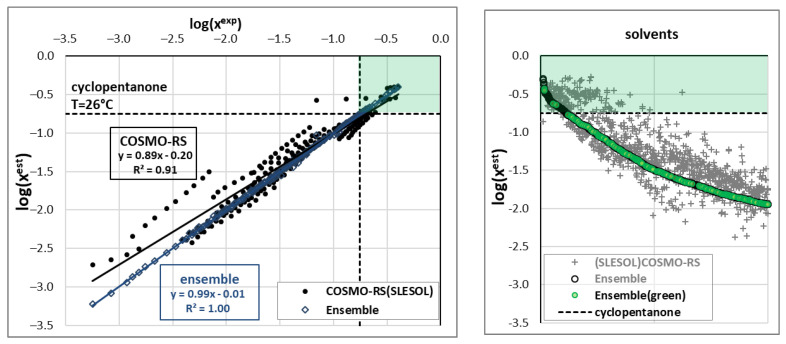
The solubility of benzenesulfonamide studied both experimentally and theoretically. The right panel compiles computed solubilities for screened solvents sorted according to ensemble-predicted values. The green rectangles represent the target zone of higher solubility compared to cyclopentanone.

The main reason for ensemble model development was to explore the solvent space, with the goal of discovering highly efficient and environmentally friendly dissolution media. The collection of available BSA solubility data indicated that, prior to our measurements, ketones such as cyclopentane or cyclohexanone were considered the solvents with the highest solubilizing potential. However, this perception was altered by the data presented in Figure 1, which demonstrates that DMSO and DMF are even better solvents. In this context, a significant and practically important question arises: is it possible to identify even more efficient solvents? This issue is addressed in Figure 4, which presents a wealth of information in a concise form. The left section of this figure illustrates the measured data, while the right panel summarizes the screening process. The black circles depict the distribution of experimental and COSMO-RS-related solubility data. It is evident that the accuracy of the prediction based on this first-principles approach is only qualitatively correct. The slope of the plotted trend indicates that COSMO-RS generally underestimates BSA solubility, although there is no linear relationship.

The dashed straight lines in Figure 4 represent BSA solubility in cyclopentanone under close-to-ambient conditions (T = 26 °C). The horizontal line represents experimental values, while the vertical line represents estimated solubility. These lines enable the identification of the region of highly efficient solvents, as indicated by the green rectangle in Figure 4. It is worth mentioning that the application of ensemble A offers almost a perfect match between the computed and measured solubilities of BSA in the studied systems. Furthermore, several points within the defined green zone suggest better solubility compared to the studied ketones, which encourages the extensive exploration of the solvent space with the aid of the developed ensemble model. The distribution of solvents, as documented by the gray circles and crosses, is highly diverse. In the window of values presented in Figure 4, only half of the tested solvents are visible, and the rest are cut off. Although the COSMO-RS method is undoubtedly a powerful and universal tool for predicting various physicochemical properties, including solubility [52,53,54], some limitations of this approach were observed in the case of BSA. Sorting the ensemble A predictions further emphasizes that screening solvents using only COSMO-RS estimation may be highly misleading. Many solvents might be identified within the green zone, but they are excluded if ensemble values are taken into account.

The presented screening resulted in the identification of 105 solvents in which the solubility potential is better than that in cyclopentanone. However, this list should be shortened using two additional criteria. The first and most important criterion is the impact on the environment. For the classification of green solvents, the environmental index (EI) proposed by the US Environmental Protection Agency (EPA) and implemented in the PARIS III application [55] was used. For the purposes of this study, all solvents with EI < 1.0 are regarded as green ones. From this perspective, cyclopentanone should be excluded, as EI(cyclopentanone) = 1.58. Conversely, two of the solvents used in this study can be included in the list of green solvents, namely, EI(DMSO) = 0.26 and EI(4FM) = 0.51. In the case of DMF, the environmental impact is higher, with EI(DMF) = 2.16, which is in line with the common notion that this solvent can hardly be regarded as eco-friendly. The green circles in Figure 4 identify acceptable cases with EI < 1.0, and the inclusion of the “greenness” criterion significantly shortened the list of solvents under consideration. Fortunately, there are still a few solvents within the green zone, namely, EI(ethanamine) = 0.81, EI(DMSO) = 0.26, EI(2,2-dimethoxy-N-methyl-ethanamine) = 0.95, EI(NMP (N-methyl-2-pyrrolidone) = 0.97, and EI(delta-octanolactone) = 0.61. It is worth mentioning that the environmental index can be reduced if aqueous mixtures are considered, which is the reason for using such binary solvents in our practice. By considering aqueous mixtures, more solvents may be included as potential candidates for eventual future experimental studies. However, it is clearly evident that the space of neat solvent reached its limits, and the probability of its extension is low. Hence, the natural continuation for the extension of the solvent space is the inclusion of multicomponent systems, which, in the case of BSA, was restricted due to the lack of experimental data.

When choosing a solvent for laboratory routines, practical factors such as price and availability are important considerations, which make up the third criterion in the selection process. Table 1 provides a comparison of the prices of various solvents with DMSO. The data indicate that DMSO is the most appropriate choice as a solvent for BSA that fulfills all the necessary requirements for extended laboratory use, including high efficiency, ecological acceptability, and affordability. The data in Table 1 suggest that there is one alternative to DMSO that also meets all criteria, which is N-methyl-2-pyrrolidone. However, this particular solvent has been found to have some ecological implications when used as a solvent. For example, NMP is classified as a hazardous air pollutant by the EPA and has been identified as a potential reproductive toxin. It is also toxic to aquatic organisms and can have negative effects on soil and groundwater quality if not handled properly. Therefore, it is advised to consider DMSO as a first-choice solvent and NMP as an alternative if, for some reason, it is not applicable. Notably, DMSO is often used as a solubilizing agent in biological activity measurements, including enzyme inhibition and in vitro assays using cell cultures [56,57,58]. The aqueous solubility of the majority of pharmaceuticals is very low, which would make it difficult to carry out this type of measurement in pure aqueous solutions. However, in the case of organic and aqueous–organic solvents, precipitation of the analyte can also occur (e.g., as a result of the sample-cooling step), which affects the reliability of the obtained results [59,60]. The selection of the appropriate solvent and appropriate concentrations limited by the solubility at a given temperature are not trivial issues and affect the accuracy of pharmacological activity measurements. This indicates the particular importance of temperature–solubility profile determination.

## 3. Materials and Methods

### 3.1. Materials

**Figure 5:**
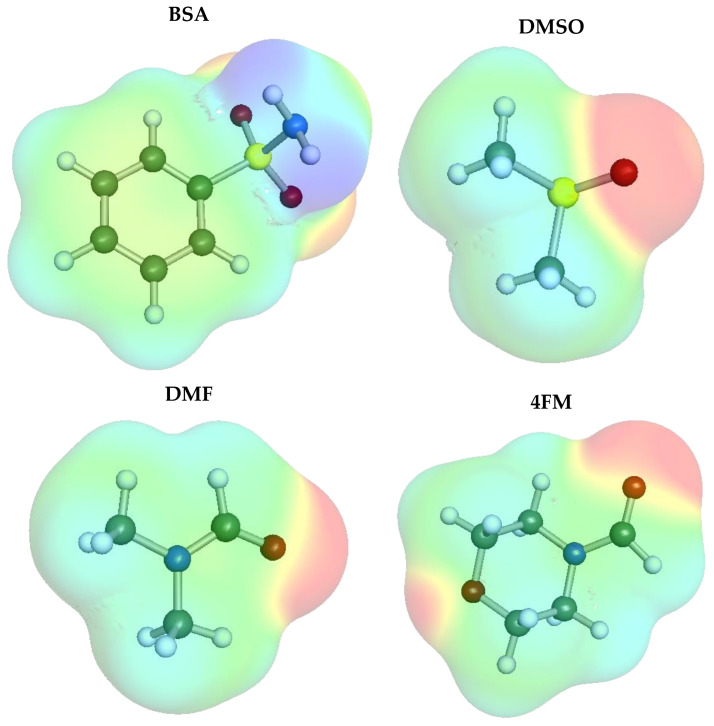
The structures, together with electron charge densities, of benzenesulfonamide and organic solvents used in this study.

Benzenesulfonamide (BSA, CAS Number: 98-10-2, MW = 157.19 g/mol) was obtained from Sigma Aldrich (Saint Louis, MO, USA), and its purity was listed as ≥98%. The three solvents, namely, Dimethylformamide (DMF, CAS Number: 68-12-2), Dimethyl sulfoxide (DMSO, CAS Number: 67-68-5), and 4-Formylmorpholine (4FM, CAS Number: 4394-85-8), were similarly supplied by Sigma Aldrich (Saint Louis, MO, USA) and also had a ≥99% purity according to the supplier. The above chemicals were used in the study without any initial procedures. The structures of the considered compounds, together with their electron charge densities, are presented in Figure 5.

### 3.2. Solubility Measurements

In order to determine the solubility of BSA in the studied solvents, excess amounts of it were added to test tubes pre-filled with a particular solvent or a binary mixture of this solvent and water in different molar proportions. Saturated solutions prepared in this manner were placed in an ES-20/60 Orbital Shaker Incubator from Biosan (Riga, Latvia) and incubated for 24 h at different temperatures. Four temperatures were used in the range from 25 °C to 40 °C with 5 °C intervals. The temperature in the incubator was adjusted with 0.1-degree accuracy with a 0.5-degree variance in a 24 h cycle. Simultaneously, the samples were mixed at 60 rev/min. In the next stage, the filtration of the samples took place, utilizing syringes equipped with PTFE filters with 0.22 µm pore size. Since the temperature difference between the measured solutions and used instruments can possibly lead to precipitation, all of the used test tubes, pipette tips, syringes, and filters were initially heated. This was carried out by placing them in the same incubator as the samples and heating them at exactly the same temperature prior to the handling of the samples. This was particularly important in the case of elevated temperatures, as the difference between, e.g., 40 °C and room temperature is quite extensive. Then, small amounts of the obtained filtrate were diluted in different test tubes containing methanol and measured spectrophotometrically. In order to determine the mole fractions of BSA in the samples, their density was also measured by weighing a 1 mL volume of each of the solutions, taken by using an Eppendorf Reference 2 pipette (Hamburg, Germany), in 10 mL volumetric flasks. The systematic error of the pipette was 6 μL. The precision of the RADWAG (Radom, Poland) AS 110.R2 PLUS analytical balance that was used in this study was 0.1 mg. The A360 spectrophotometer from AOE Instruments (Shanghai, China) was used for solubility determination. The spectra were recorded in the 190 nm to 400 nm wavelength range with a 1 nm resolution. Methanol was used for both the dilution of the samples and the initial calibration of the spectrophotometer. The analytical wavelength was determined to be 264 nm, and the absorbance of the samples at this wavelength was used for the determination of the BSA concentration in the samples and the subsequent calculation of its mole fractions. Three separate measurements were used, and the resulting values were averaged. The calibration curve for BSA was generated by preparing successive dilutions of an initial stock solution and performing spectrophotometric measurements of the resulting solutions with decreasing concentrations. The molar concentrations of measured solutions were 3.19 × 10^−3^, 2.84 × 10^−3^, 2.46 × 10^−3^, 2.13 × 10^−3^, 1.88 × 10^−3^, 1.60 × 10^−3^, 1.26 × 10^−3^, 9.53 × 10^−4^, 5.70 × 10^−4^, and 4.80 × 10^−4^ M). The relationship between the absorbance values at 264 nm and the solution concentration was described by the linear equation A = 470.86 × C + 0.0093 (A—absorbance; C—molar concentration), with a determination coefficient R^2^ equal 0.9996, denoting high linearity.

### 3.3. Instrumental Analysis of Solid Residues

The Fourier transform infrared spectroscopy (FTIR) and differential scanning calorimetry (DSC) techniques were used to analyze the solid residues remaining after the solubility determination. Prior to this analysis, the samples were removed from test tubes and dried in the air. The FTIR spectra were recorded using a Spectrum Two spectrophotometer from Perkin Elmer (Waltham, MA, USA) equipped with an attenuated total reflection (ATR) device. A wavenumber range of 450–4000 cm^−1^ was used. The DSC analysis was conducted using a DSC 6000 calorimeter from PerkinElmer (Waltham, MA, USA). A heating rate of 5 K/min was used, with a 20 mL/min nitrogen flow providing the inert atmosphere. The samples were placed in standard aluminum pans, and the initial calibration of the apparatus was conducted using indium and zinc standards. The calibration errors related to the temperature and enthalpy of fusion values were 0.03% and 2%, respectively.

### 3.4. Solubility Dataset

The solubility of benzenesulfonamide (BSA) has only been studied in neat solvents, with a limited dataset comprising 190 measurements. To build a model for predicting BSA solubility, the dataset was split into three subsets: a training set (70% of the data) and two additional sets for testing and validation (30% of the data). During training, these subsets were hidden and only used for evaluation after model development. A previous study by Li et al. [28] presented the measured BSA solubility in various neat solvents and solvent mixtures, including alcohols (methanol, ethanol, n-propanol, isopropanol, n-butanol, isobutanol, n-pentanol, and isopentanol), esters (ethyl formate, methyl acetate, and ethyl acetate), and ketones (acetone, cyclopentanone, and cyclohexanone), as well as acetonitrile and dichloromethane. This study expanded the solvent space by including new measurements in 4-FM, DMF, DMSO, and water.

### 3.5. Model Development

The problem of the solubility prediction was solved using the Python code developed for the purpose of this study by the hyperparameter tuning of 36 regression models utilizing a variety of algorithms, including linear models, boosting, ensembles, nearest neighbors, neural networks, and also some other types of regressors. The search for their optimal parameters was carried out using the Optuna study, which is a freely available Python package for hyperparameter optimization [61]. The collection of the tuned models was formulated after 5000 minimization trials using TPE (Tree-structured Parzen Estimator) as a sampler of the search algorithm. TPE is a computationally efficient model-based optimization algorithm that uses a probability density function to model the relationship between hyperparameters and performance metrics. To evaluate the performance of each regression model, a new custom score function was developed that combines multiple metrics to take into account both the model’s accuracy and ability to generalize. The actual mathematical formula used for the loss computation is the following:

(1) $${loss}_{train}={MSE}_{train}^{LC,train}+\left|{MSE}_{train}^{LC,train}-{MSE}_{train}^{LC,test}\right|\;+{MSE}_{train}(1+100\cdot N_{traint}^{pos}+10\cdot N_{train}^{out})$$

where all terms were computed on the training dataset. The last term comprises the value of the mean squared error (${MSE}_{train}$) between the predicted and actual values of the target variable and two penalties on the number of positive values ($N_{train}^{pos})$ and outliers ($N_{train}^{out}$). The first penalty is associated with the formally acceptable predicated values since the models were trained against the values of solubility expressed as the logarithm of the mole fraction and, as such, should always be positive. The latter penalty directs the acceptance of models with as few as possible outlying data points, defined as 3 times higher than the standard deviation. The first two terms in Equation (1) were obtained from the learning curve analysis (LCA) of the scikit-learn 1.2.2 library [51] and provide information on the model’s performance for different training set sizes. It is worth mentioning that LCA utilizes cross-validation (CV), which was set here to a 5-fold CV of the training dataset. The ${MSE}_{train}^{LC,train}$ and ${MSE}_{train}^{LC,test}$ values were obtained from the learning curve analysis, which provides information on the model’s ability to generalize to new, unseen data. The learning curve analysis (LCA) was performed using the sklearn.model_selection.learning_curve function from the scikit-learn library [51]. Since LCA can be computationally expensive, here, only two-point computations were performed by including 50% to 100% of the total data. The final model’s assessments via LCA were conducted using 20-point computations. The values included in the custom loss correspond to the mean MAE values obtained on the largest training set size. Hence, such a custom loss function combines the two types of components providing information on the model’s accuracy and ability to generalize to new, unseen data. Overall, this approach is regarded as a robust and reliable solubility prediction model that can be used for various applications and screening for new solvents.

The final performance of all models was evaluated using loss values characterizing test and validation subsets. The ensemble model (EM) was defined by the inclusion of the subset of regression models with the lowest values of both criteria, and the final predictions were averaged over selected models.

### 3.6. Molecular Descriptors

The selection of appropriate molecular descriptors for machine learning models is a critical step in model development [62]. For the ensemble model developed in this study, only molecular descriptors computable from the molecular structure were selected, as the main goal was to preselect the most suitable green solvents for solubility measurements. In addition, since experimental data are temperature-dependent, commonly used descriptor generators were excluded. Instead, the COSMO-RS quantum chemical approach [63] implemented in the COSMOtherm software, (BIOVIA COSMOtherm 2021 (build: 21.0.0)) [64] was used to estimate the physicochemical characteristics of the system, including solubility, activities, chemical potential, intermolecular interaction, and others. The use of COSMO-RS descriptors has been documented in previous studies and has shown good performance in predicting solubility and other properties. Initially, hundreds of potential descriptors were considered, and a selection process was applied to identify the final set of descriptors used for model training. The selection criteria included a high correlation with experimental solubility, sufficient variability, and a lack of inter-correlations [65]. This restricted the selection to four descriptors, namely, the estimated solubility with COSMO-RS, log(x^est^); the relative value of the infinite dilution activity coefficient (IDAC), $∆ln\left(\gamma_{12}^{\infty }\right)$; the relative value of the third σ–moment; and the mean value of the solvent σ–potential for the highest percentile of the positive section of σ-distribution. The first descriptor was computed using the SLESOL approach for solving the SLE equation defined by COSMOtherm software. This is computationally more expensive than the commonly applied iterative procedure but can help avoid the occasional failure of the latter algorithm in not providing conclusive solubility.

For the successful application of this procedure, fusion data need to be provided, as COSMO-RS is unable to treat solids. For BSA, the following values were used: heat of fusion, ${∆H}_{fus}^{}$ = 25.6 J/mol, and melting point, *T_m_* = 425.4 K, as averaged over values reported in the literature [28,66]. Unfortunately, there are no data about the experimental heat capacity change upon melting, ${∆C}_{p,fus}^{}$, which is required for the thermodynamic characteristics of the solid–liquid equilibrium via the fundamental equation:

(2) $$ln{(\gamma_{i}\cdot x}_{1}^{id})=\frac{∆H_{fus}^{}}{R}\left(\frac{1}{T_{m}}-\frac{1}{T}\right)-\frac{1}{RT}\int_{T_{m}}^{T}∆C_{p,fus}dT+\frac{1}{R}\int_{T_{m}}^{T}\frac{∆C_{p,fus}}{T}dT$$

where the left side defines the solute activity ${a_{i}{=\gamma }_{i}\cdot x}_{1}^{id}$. The application of this equation for any solid solute is often simplified by assuming the temperature independence of ${∆C}_{p,fus}^{}$. There are two common alternatives, which suffer from different inaccuracies depending on the studied system. The crudest simplification ignores heat capacity by setting ${∆C}_{p,fus}^{}$= 0, which is often quite acceptable [67,68]. Alternatively, one can assume ${∆C}_{p,fus}^{}{\approx S}_{fus}^{}$ = $∆H_{fus}^{}/T_{m}$.

**Figure 6:**
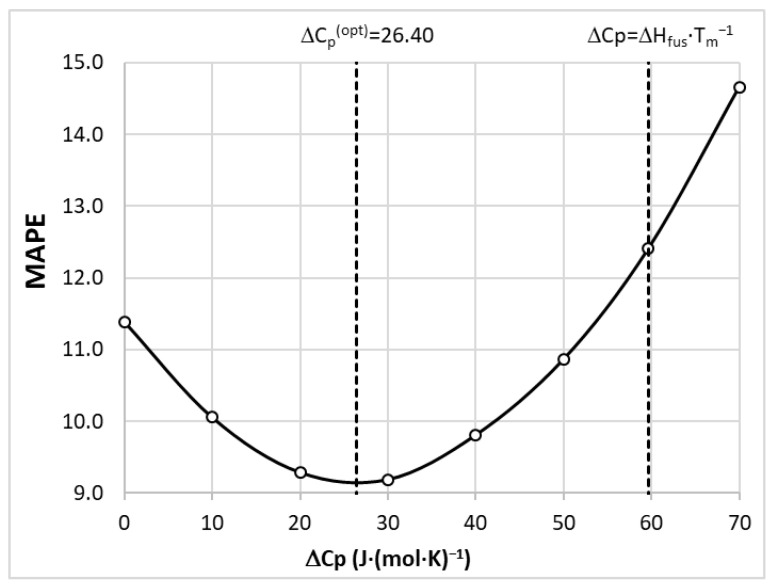
Quantitative characterization of the accuracy of the solubility calculation depending on the value of Δ𝐶_𝑝,𝑓𝑢𝑠_.

Since these simplifications can introduce system-dependent inaccuracies, for the purpose of this paper, the ${∆C}_{p,fus}^{}$ value was optimized for minimizing MAPE (mean average percentage error). The results of the performed optimization are presented in Figure 6. It is interesting to note that in the case of BSA, the COSMO-RS-derived solubility values are characterized by MAPE equal to 11.4% and 12.4% after assuming ${∆C}_{p,fus}^{}$ = 0 and ${∆C}_{p,fus}^{}{\approx S}_{fus}^{}$, respectively. The setting of ${∆C}_{p,fus}^{}$ = 26.4 J/(mol·K) reduces the MAPE values down to 9.2%. This is not a very spectacular gain, especially because one can find cases for which the percentage error is higher than 50%, irrespective of the value of the heat capacity change. Nevertheless, the computed solubility is a very important molecular descriptor, which has the highest contributions to all tuned models, as found via molecular descriptor importance analyses performed for every regressor.

The relative IDAC values were computed from directly available infinite dilution coefficients:

(3) $$∆ln\left(\gamma_{12}^{\infty }\right)=ln\left(\gamma_{1}^{\infty }\right)-ln\left(\gamma_{1}^{\infty }\right)$$

The COSMO-RS theory introduced the Taylor-series expansion of the *σ*-potential:

(4) $$M_{i}^{BSA}=\int p^{BSA}\left(\sigma \right)\cdot \sigma^{i}d\sigma$$

with contributions named *σ*-moments. The zero-order *σ*-moment, $M_{i=0}^{BSA}$, is simply the molecular area of BSA; the first *σ*-moment, $M_{i=1}^{BSA}$, is the negative of the total charge of the compound; the second *σ*-moment, $M_{i=2}^{BSA}$, is correlated with the screening charge of the system; and the third *σ*-moment of the system, $M_{i=3}^{BSA}$, is a measure of the skewness of the *σ*-profile of BSA in the mixture. The fourth parameter originated from the *σ*-potential computations of a solute in the solvent. As was previously documented [69], it is practical to reduce the data from a 61-point dataset to between −0.03 and +0.03 by averaging over 0.01 segments. The last interval between sigma potentials of solvents happened to be a quite useful descriptor and was used for model development.

All descriptor values were extracted just from the output generated during solubility, activity, or *σ*-potential computations using COSMOtherm [64]. It is also essential to add that the full conformational analysis of all structures was carried out using the COSMOconf program [70] on the level standardized for the highest parametrization available, namely, BP_TZVPD_FINE_21.ctd.

## 4. Conclusions

This study investigated the solubility of benzenesulfonamide as a model compound, which is a precursor for many active pharmaceutical ingredients. The limited experimental data on benzenesulfonamide solubility were extended by providing the results of new measurements in binary aqueous solutions of DMSO, DMF, and 4FM. This not only extended the set of measured BSA solubility but also changed the perspective of solvent effectiveness, suggesting that not only ketones, such as cyclopentane or cyclohexanone, are very efficient solubilizers, but heteroatom-containing molecules are also worth considering. Indeed, the choice of such solvents was found to be appropriate, as DMSO and DMF turned out to be more efficient compared to ketones, which were previously considered the most effective. So far, the presented results of new measurements are the only available data for binary solvent mixtures used for BSA dissolution. No solvation effect was observed, as in the whole range of binary mixture compositions, water is an efficient anti-solvent for each of the studied media.

Thirty-six regression models were trained on all available experimental data points (N = 190) for tuning the whole hyperparameter space using in-house-developed Python code. The central part of the optimizations, performed using facilities of the Optuna study, was a novel scoring function defined for considering not only accuracy but also the bias–variance tradeoff via learning curve analysis. The idea behind this was not only to achieve high accuracy of back-computed data used for model training but also to ensure the predictability of the model by reducing its vulnerability to overfitting. To the authors’ best knowledge, this approach is unique and novel in the development of solubility models. An ensemble of the top regression models based on the scoring function made reliable predictions by comparing the values and standard deviations. The ensemble very accurately back-calculated experimental data and predicted solubility in 2067 potential solvents. The final predictions were made using an ensemble of regression models identified based on the values of the scoring function estimated for unseen data during the training stage. The reliability of the predictions was ensured by comparing the solubility values and the corresponding standard deviations of two ensembles: one with seven regressors and the other extended by three additional models. The comparable predictions demonstrated that the smaller set of models was sufficient.

The molecular descriptors used for model training were derived using the COSMO-RS approach, providing many temperature- and composition-dependent physicochemical properties. Among many available features, the computed solubility values were found to possess the most significant contribution to all regressors. This is despite the fact that the computed absolute values only qualitatively reproduce the experimental solubility of BSA. For the highest accuracy, the value of the heat capacity change upon melting was optimized for the minimization of MAPE. This led to the interesting observation that neither of two the most common assumptions, ${∆C}_{p,fus}^{}$ = 0 and ${∆C}_{p,fus}^{}{\approx S}_{fus}^{}=59.7J/(mol\cdot K)$, are the best choice for BSA solubility computations. Instead, the value corresponding to 26.4 J/(mol·K) was found to be more accurate. Although the MAPE values were reduced to 9.2%, one can find cases for which the percentage error is higher than 50%. It is important to note that despite these shortcomings, the computed solubility value using the COSMO-RS approach can be regarded as a very valuable molecular descriptor due to its high importance for regressor hyperparameter tuning.

## Figures and Tables

**Table 1:** The compilation of selected properties identified from solvent screening.

Solvent Name	CAS	EI	Relative Price	log(x_BSA_^pred^)
Ethanamine	[109-85-3]	0.81	6.9	−0.43
DMSO	[67-68-5]	0.26	1.0	−0.45
2,2-Dimethoxyethylmethylamine	[122-07-6]	0.95	7.3	−0.63
N-Methyl-2-pyrrolidone	[872-50-4]	0.97	0.3	−0.67
Delta-octanolactone	[698-76-0]	0.61	434.1	−0.77

## Data Availability

All data supporting the reported results are available on request from the corresponding author.

## Supplementary Materials

The following supporting information can be downloaded at https://www.mdpi.com/article/10.3390/molecules28135008/s1. (a) Document files: S1. Benzenesulfonamide solubility values in aqueous–organic binary solvents containing DMSO, DMF, and 4FM (4-formylmorpholine); S2. Instrumental analysis of solid residues obtained after shake-flask solubility determination procedure; S3. Regression model performance. (b) The Excel file models.xlsx provides values of the experimental and predicted solubilities, all molecular descriptors, and details of hyperparameters tuned for every regressor and their performance (both ensembles A and B). (c) The Excel file predictions.xlsx provides molecular descriptors and predictions made for all screened solvents.
