# Supplementary material for: Finding the Right Solvent: A Novel Screening Protocol for Identifying Environmentally Friendly and Cost-Effective Options for Benzenesulfonamide
Source: Molecules. 2023 Jun 26;28(13):5008. doi: 10.3390/molecules28135008 (PMC10343355; doi:10.3390/molecules28135008)
Supplement: Supplementary file 1 [file molecules-28-05008-s001.zip › BSA_SM.docx]

**Supplementary Materials**

**Finding the Right Solvent: A Novel Screening Protocol for Identifying Environmentally Friendly and Cost-Effective Op-tions for Benzenesulfonamide**

Piotr Cysewski*, Tomasz Jeliński, Maciej Przybyłek

*Department of Physical Chemistry, Pharmacy Faculty, Collegium Medicum of Bydgoszcz, Nicolaus Copernicus University in Toruń, Kurpińskiego 5, 85-096 Bydgoszcz, Poland*

**Correspondence: piotr.cysewski@cm.umk.pl*

**Table of content**

[**S1. Benzenesulfonamide solubility values in aqueous-organic binary solvents containing DMSO, DMF and 4FM (4-formylmorpholine).** 2](#_Toc137618928)

[**Table S1**. The experimentally determined benzenesulfonamide (BSA) solubility values (X^exp^) in DMSO, DMF, 4FM and their aqueous mixtures. 2](#_Toc137618929)

[**S2. Instrumental analysis of solid residues obtained after shake-flask solubility determination procedure** 3](#_Toc137618930)

[**Figure S1.** DSC thermograms recorded for pure benzenesulfonamide and the solid residues collected after shake-flask solubility determination procedure. 3](#_Toc137618931)

[**Figure S2.** FTIR spectra recorded for pure benzenesulfonamide and the solid residues collected after shake-flask solubility determination procedure. 4](#_Toc137618932)

[**S3. Regression model performance** 5](#_Toc137618933)

[**Table S2.** Compilation of the model analysis in terms of distributions of applicability domain and learning curve analysis. For all models included in the ensemble A. The size of points corresponds to values of standardized error (se). 5](#_Toc137618934)

# **S1. Benzenesulfonamide solubility values in aqueous-organic binary solvents containing DMSO, DMF and 4FM (4-formylmorpholine).**

## **Table S1**. The experimentally determined benzenesulfonamide (BSA) solubility values (X^exp^) in DMSO, DMF, 4FM and their aqueous mixtures.

| **BSA+DMSO+water (solubility,** **X^exp^ × 10^3^)** | | | | |
| --- | --- | --- | --- | --- |
| **x_2_*** | **298.15 K** | **303.15 K** | **308.15 K** | **313.15 K** |
| **0.0** | 0.56 ± 0.03 | 0.83 ± 0.01 | 1.17 ± 0.04 | 1.53 ± 0.03 |
| **0.2** | 35.84 ± 1.56 | 40.39 ± 0.41 | 45.37 ± 0.84 | 50.66 ± 1.03 |
| **0.4** | 136.28 ± 5.21 | 153.75 ± 0.89 | 175.06 ± 5.16 | 198.55 ± 12.9 |
| **0.6** | 257.01 ± 5.42 | 279.84 ± 7.39 | 307.25 ± 8.78 | 335.53 ± 2.01 |
| **0.8** | 301.83 ± 4.01 | 324.42 ± 10.85 | 352.69 ± 7.04 | 381.96 ± 8.16 |
| **1.0** | 319.77 ± 2.83 | 342.67 ± 5.19 | 369.38 ± 9.57 | 402.31 ± 9.04 |
| **BSA+DMF+water (solubility, X^exp^ × 10^3^)** | | | | |
| **0.2** | 42.18 ± 0.83 | 46.69 ± 1.75 | 54.78 ± 1.76 | 63.21 ± 1.40 |
| **0.4** | 161.16 ± 3.97 | 179.12 ± 10.87 | 202.07 ± 2.93 | 233.72 ± 14.10 |
| **0.6** | 231.63 ± 6.50 | 253.07 ± 9.75 | 281.23 ± 1.05 | 319.28 ± 11.90 |
| **0.8** | 268.17 ± 4.27 | 286.69 ± 8.71 | 317.90 ± 8.49 | 356.68 ± 8.53 |
| **1.0** | 288.65 ± 1.99 | 308.50 ± 5.81 | 336.89 ± 13.52 | 378.60 ± 8.94 |
| **BSA+4FM+water (solubility, X^exp^ × 10^3^)** | | | | |
| **0.2** | 23.42 ± 0.86 | 32.61 ± 1.65 | 48.14 ± 1.59 | 69.19 ± 2.56 |
| **0.4** | 45.67 ± 0.33 | 68.93 ± 1.12 | 102.78 ± 3.56 | 142.57 ± 6.01 |
| **0.6** | 61.48 ± 0.85 | 101.16 ± 0.91 | 146.31 ± 6.19 | 196.38 ± 7.85 |
| **0.8** | 68.53 ± 0.63 | 118.09 ± 6.45 | 178.71 ± 6.67 | 242.26 ± 2.09 |
| **1.0** | 69.45 ± 0.49 | 132.21 ± 4.61 | 200.82 ± 7.43 | 279.66 ± 2.42 |

# **S2. Instrumental analysis of solid residues obtained after shake-flask solubility determination procedure**

## **Figure S1.** DSC thermograms recorded for pure benzenesulfonamide and the solid residues collected after shake-flask solubility determination procedure.

## **Figure S2.** FTIR spectra recorded for pure benzenesulfonamide and the solid residues collected after shake-flask solubility determination procedure.

# **S3. Regression model performance**

## **Table S2.** Compilation of the model analysis in terms of distributions of applicability domain and learning curve analysis. For all models included in the ensemble A. The size of points corresponds to values of standardized error (se).

| **Regressor** | **Results** |
| --- | --- |
| **NuSVR**  **set A** | 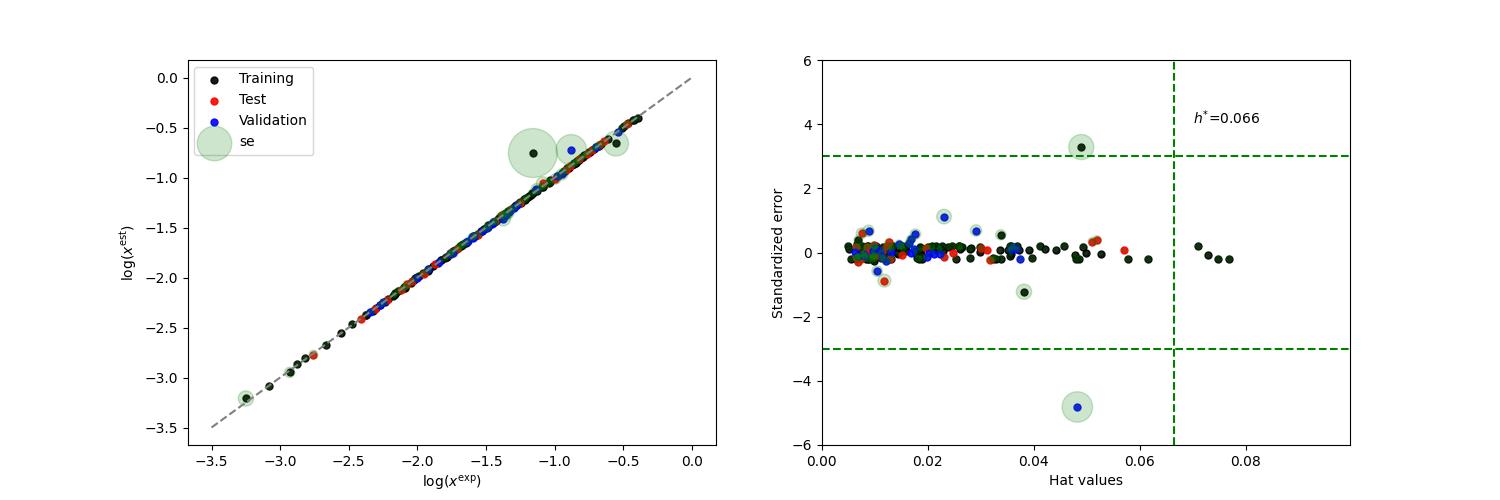  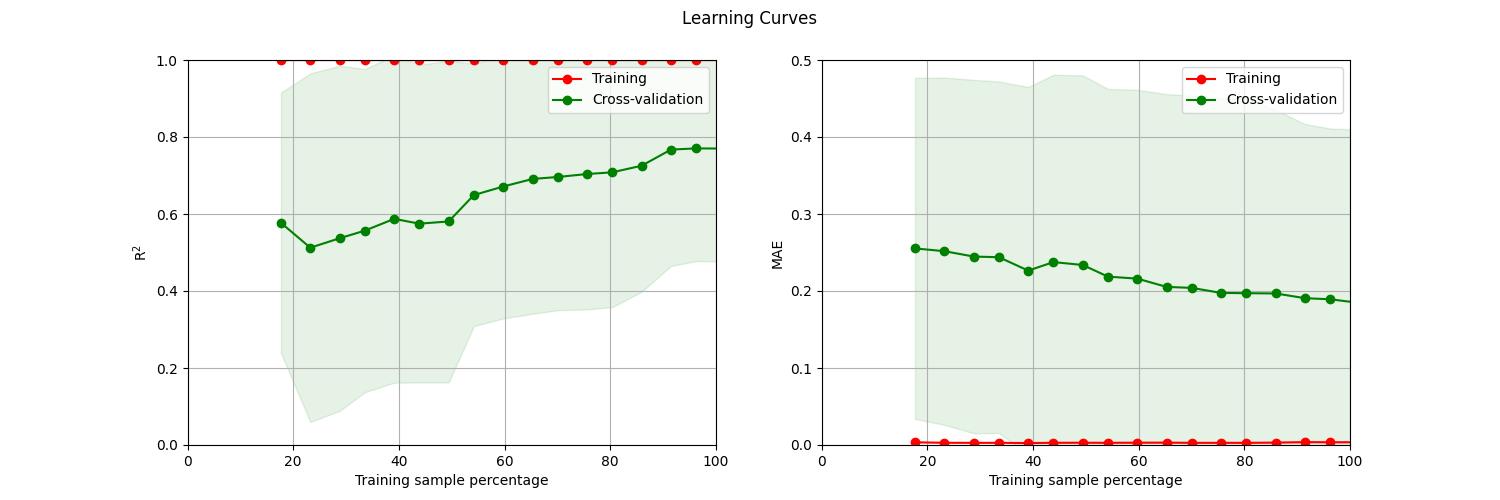 |
| **SVR**  **set A** | 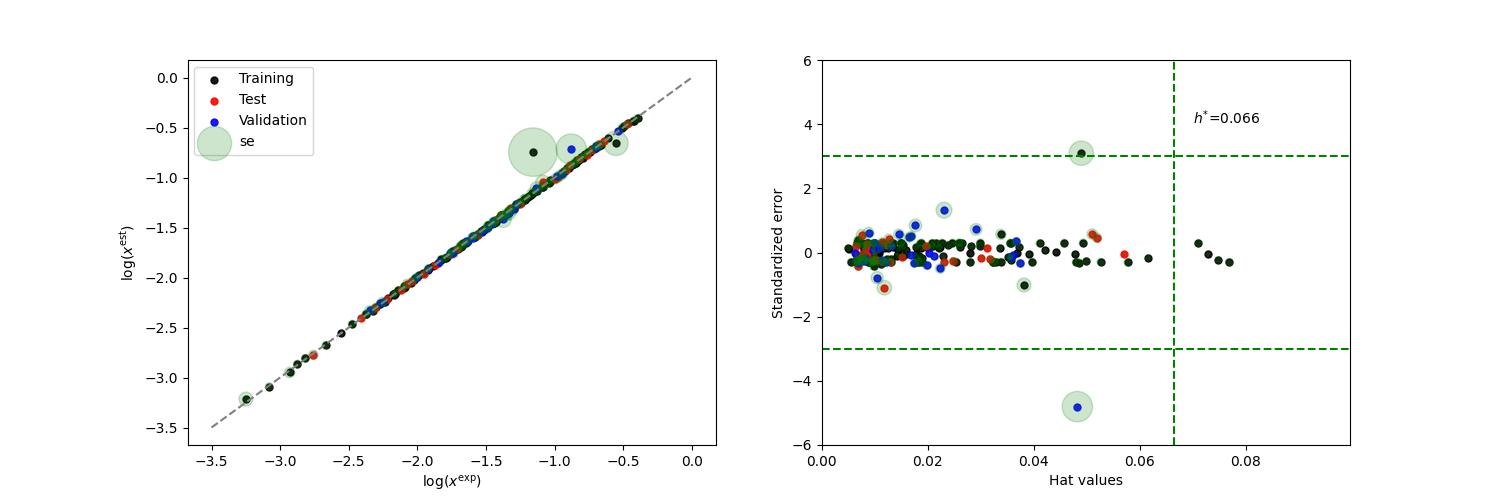  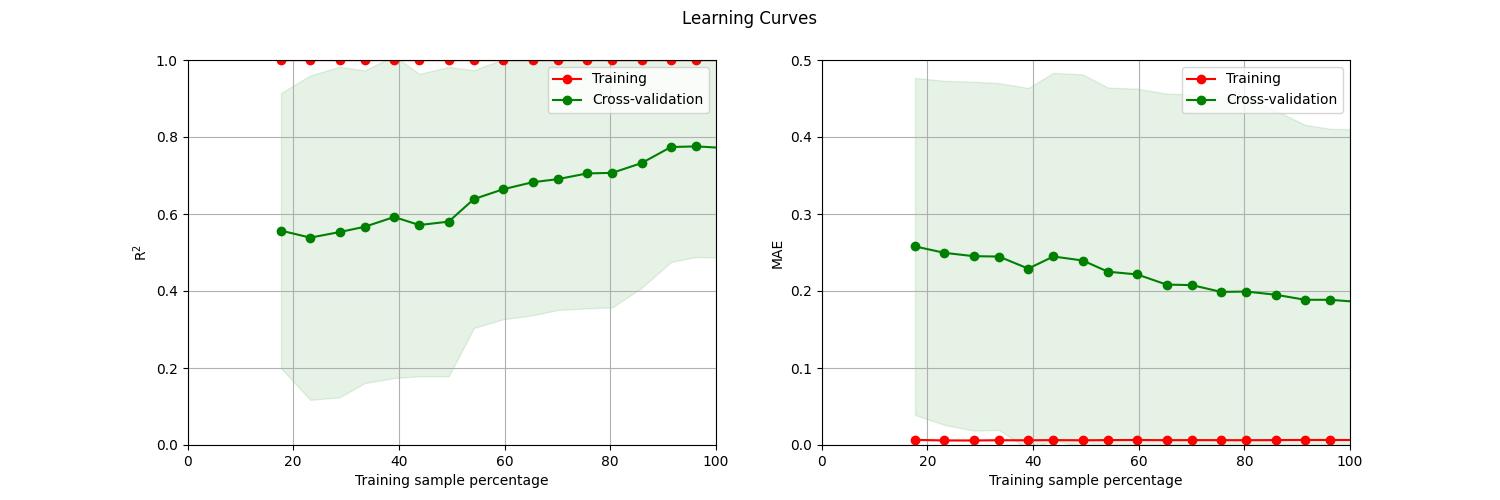 |
| **MLPRegressor**  **set A** | 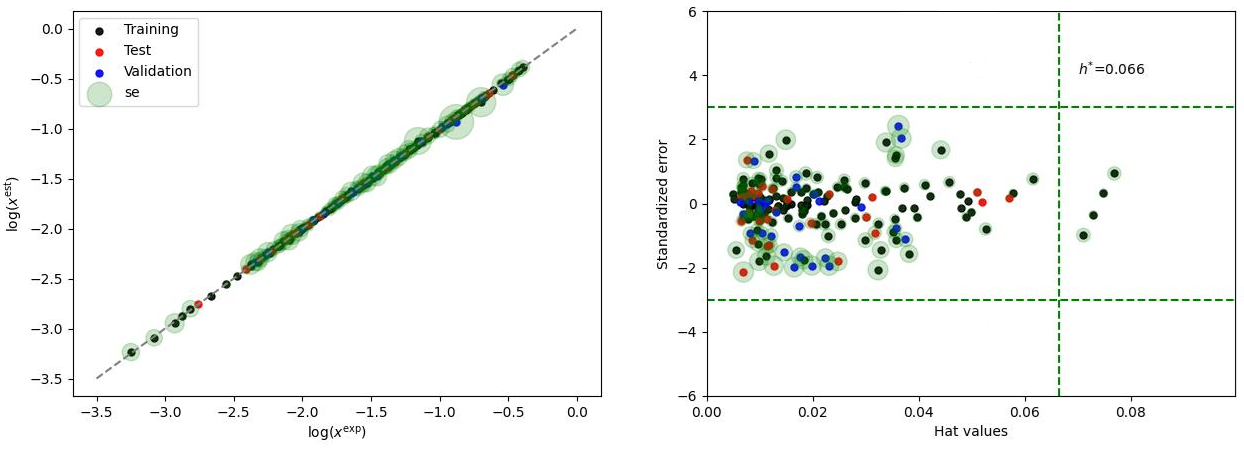  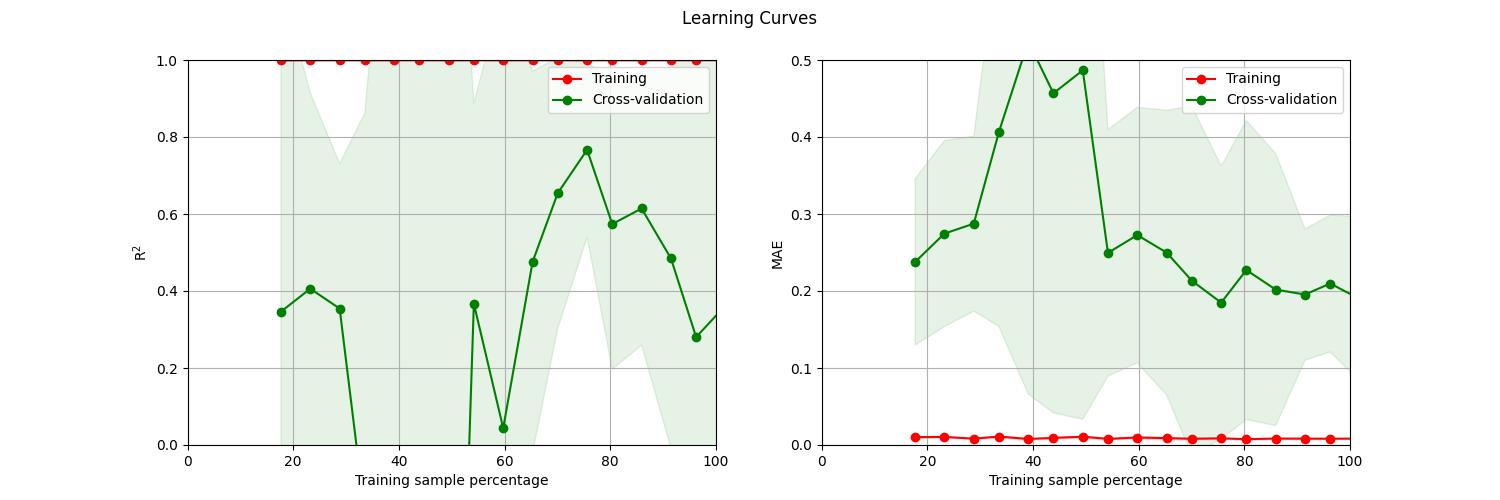 |
| **KNeighborsRegressor**  **set A** | 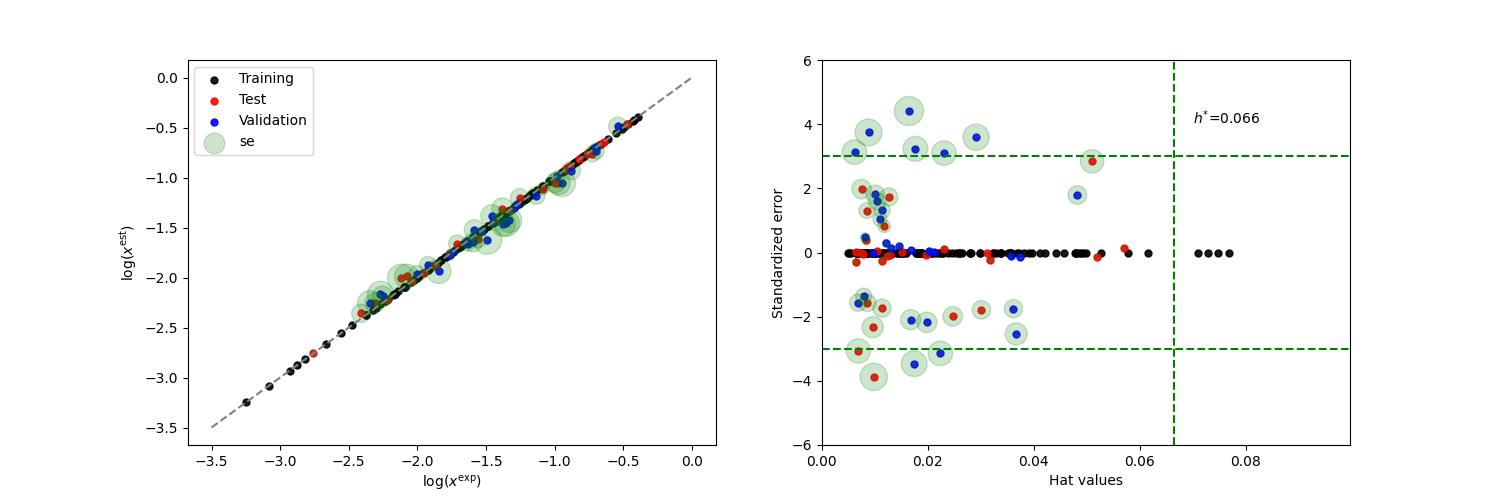  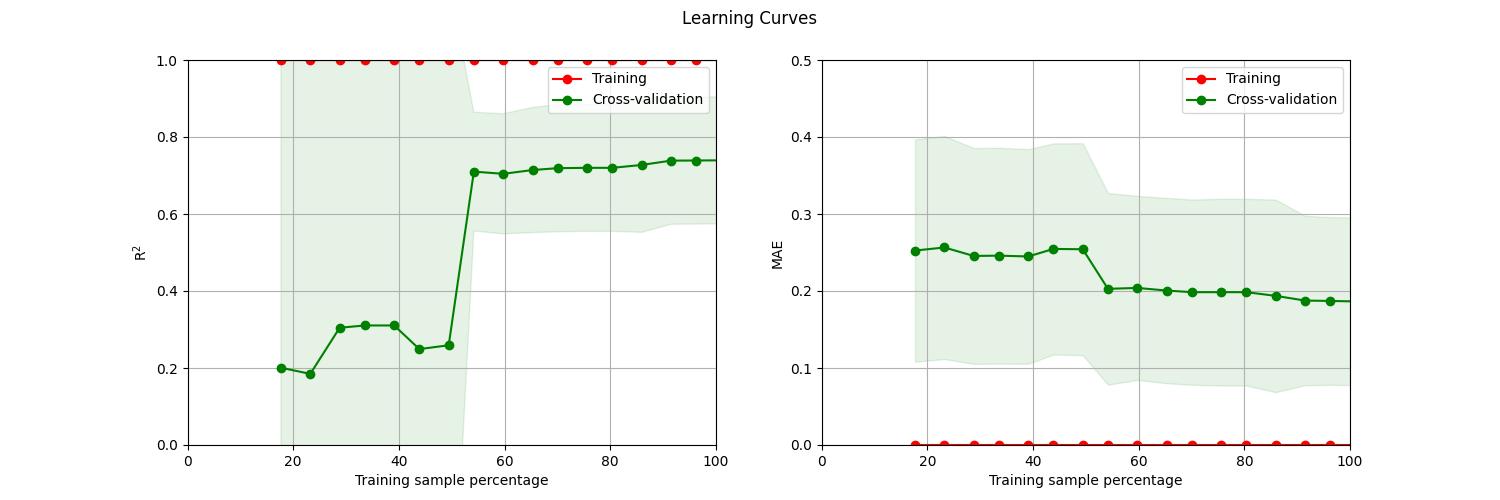 |
| **GradientBoostingRegressor**  **set A** | 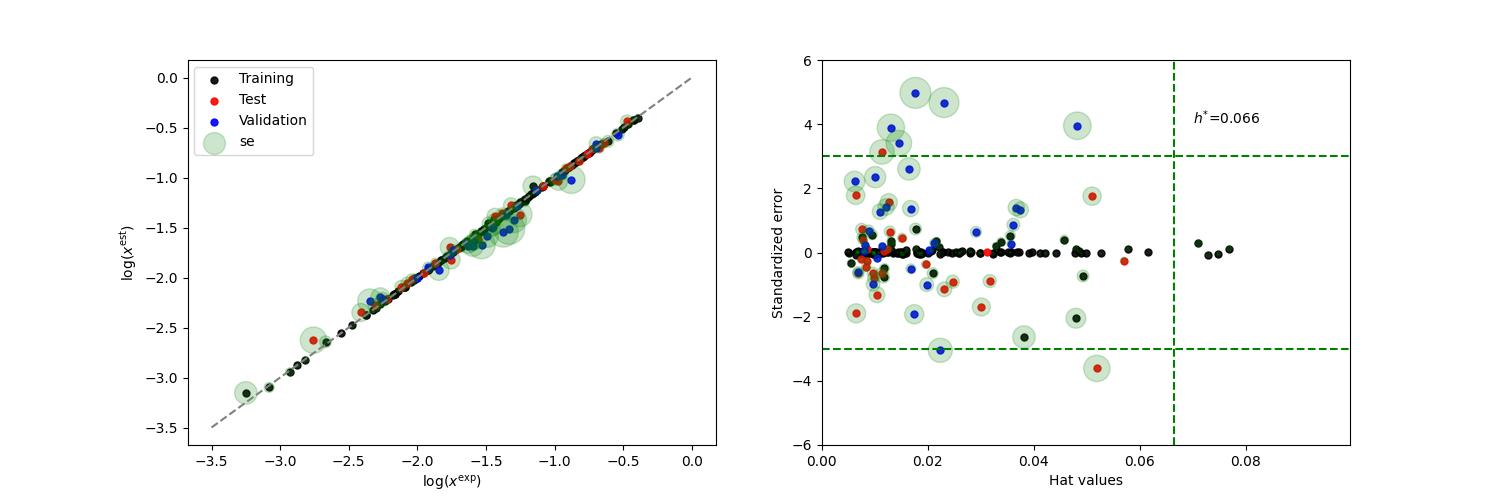  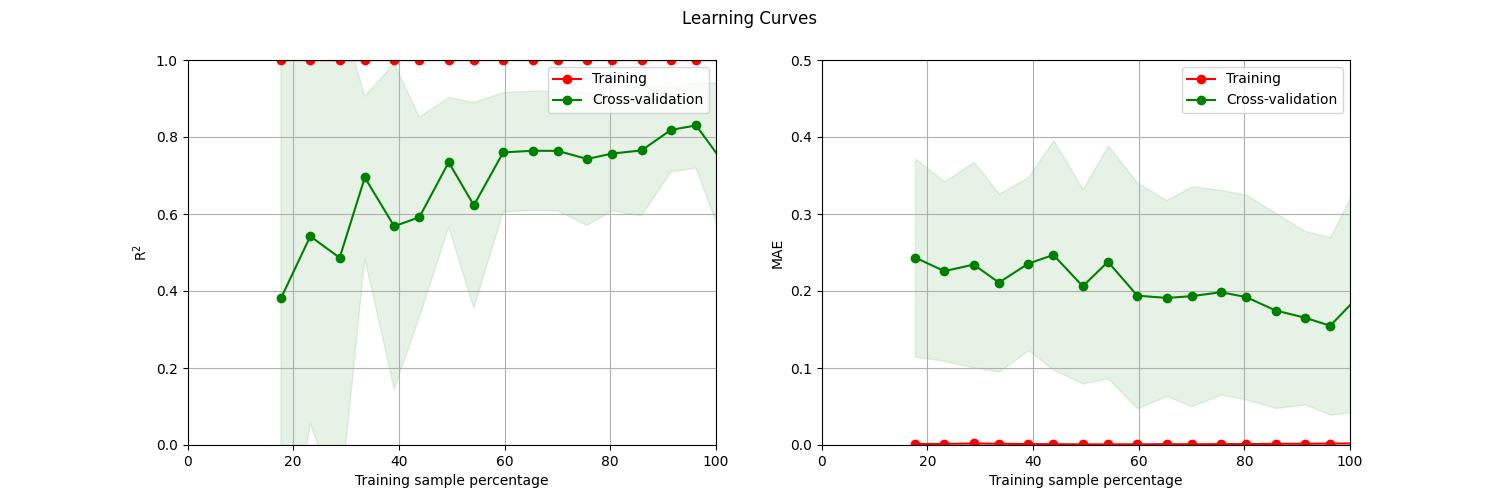 |
| **CatBoostRegressor**  **set A** | 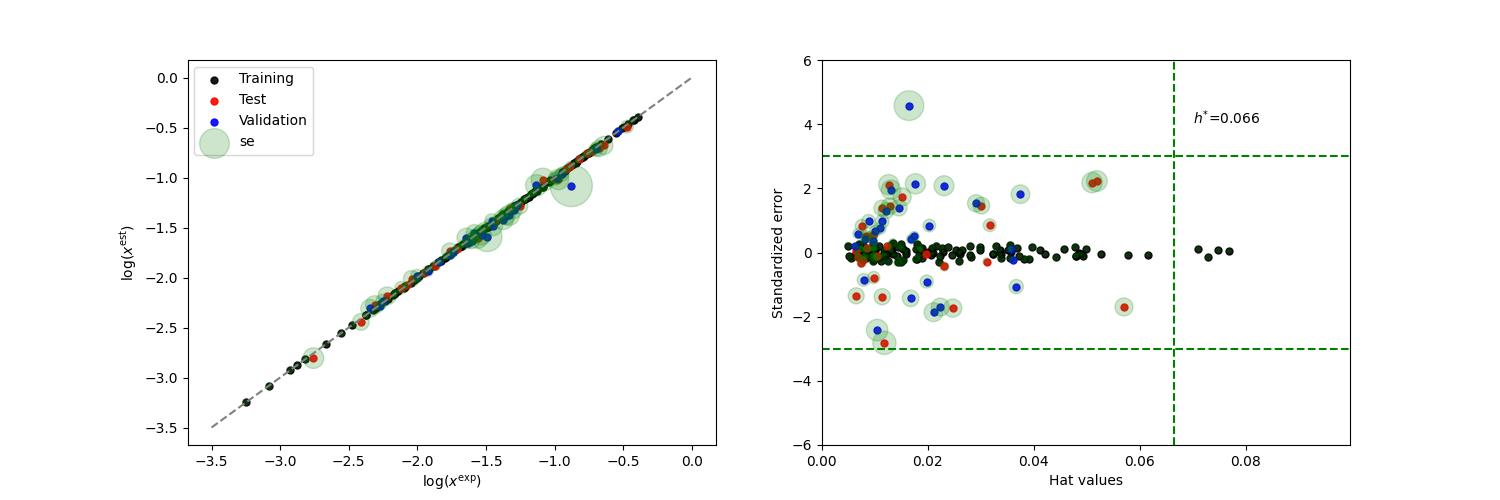  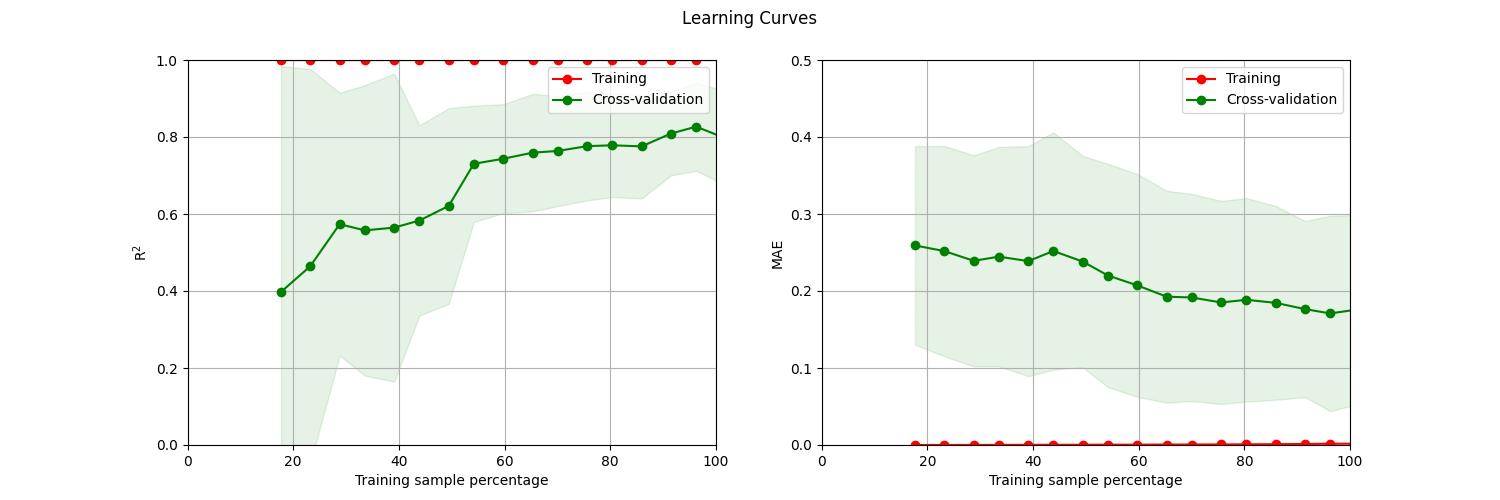 |
| **HistGradientBoostingRegressor**  **set A** | 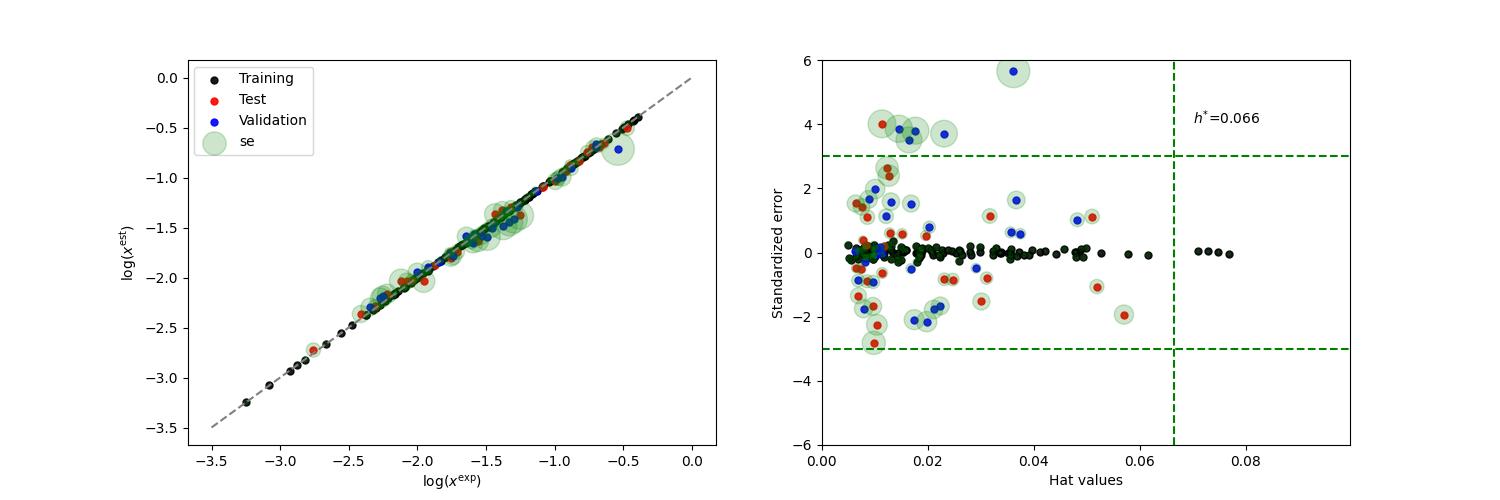  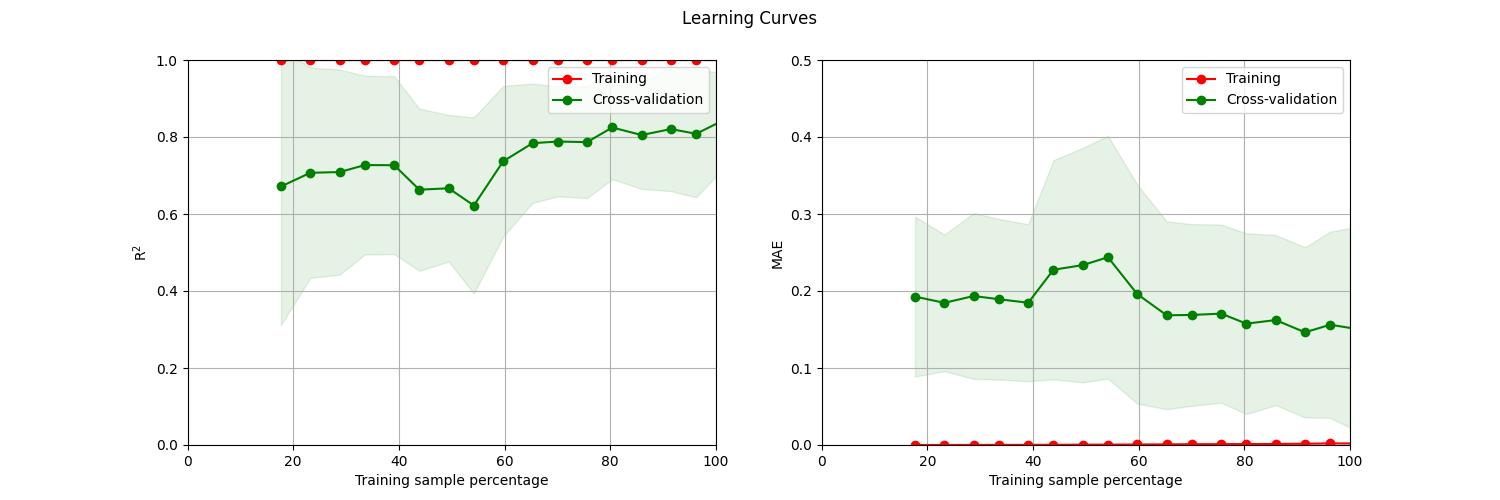 |
| **AdaBoostRegressor**  **set B** | **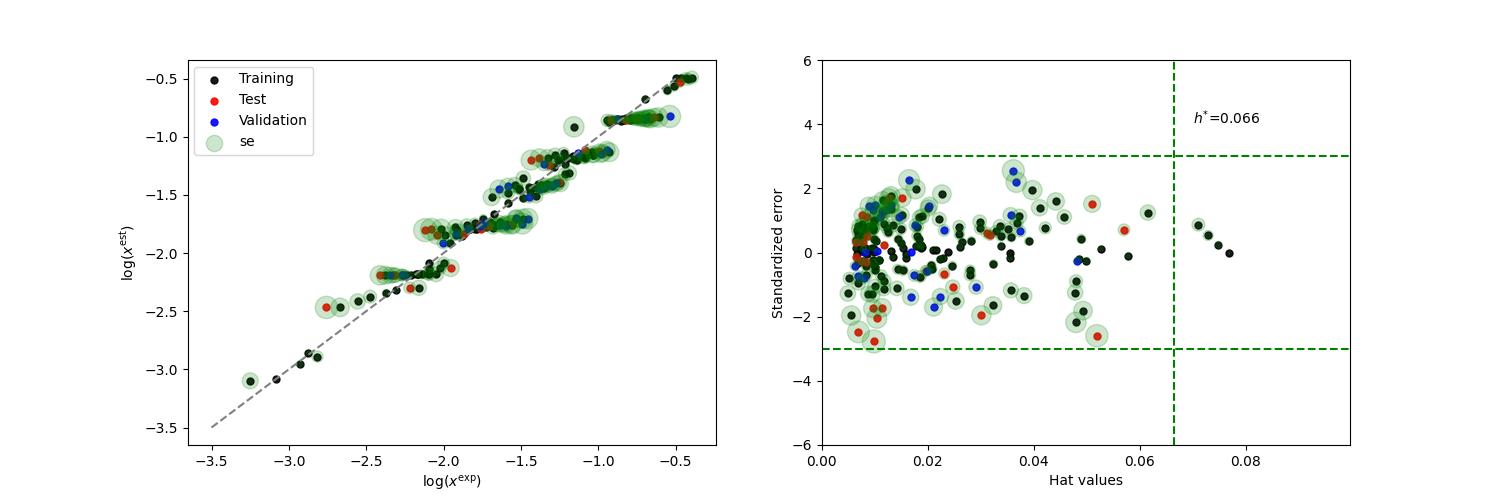**  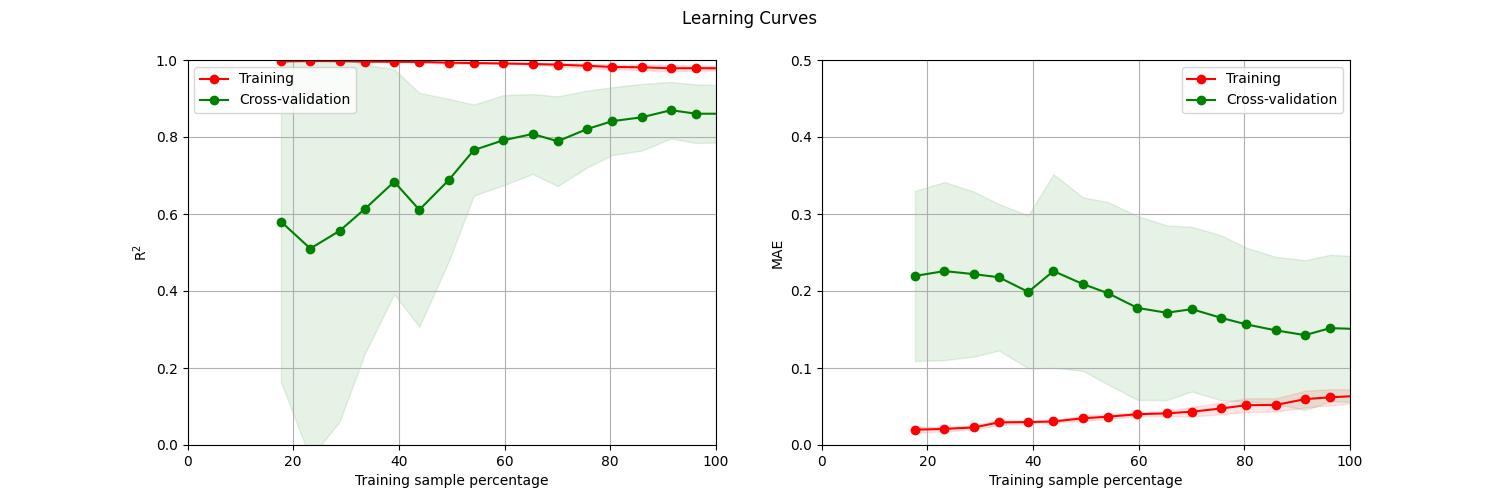 |
| **LGBMRegressor**  **set B** | 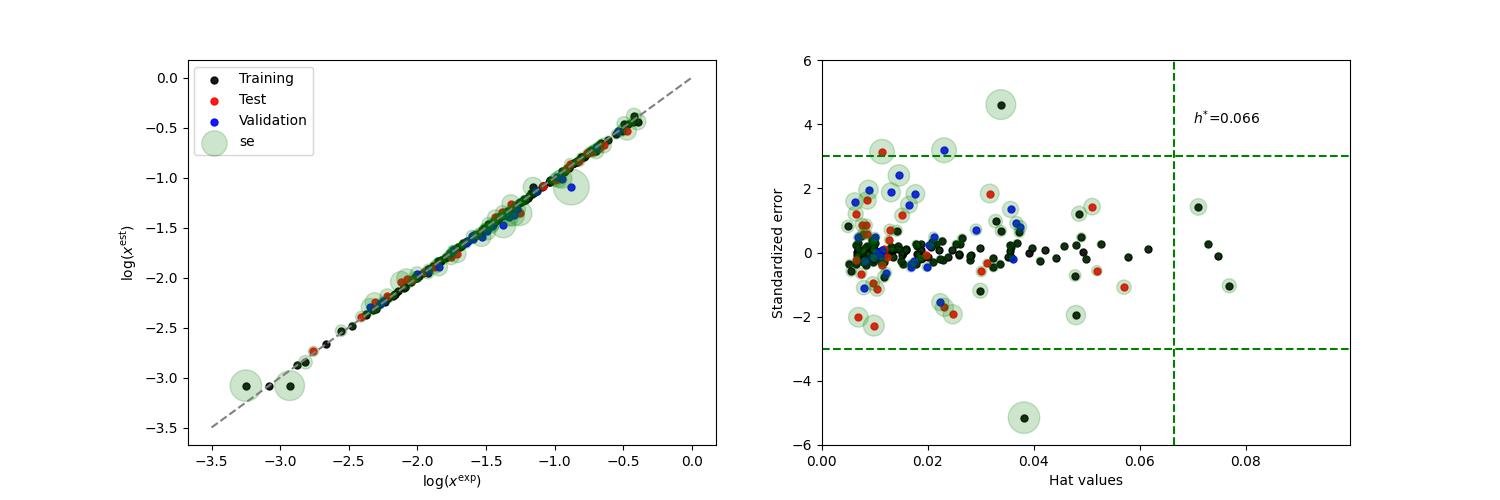  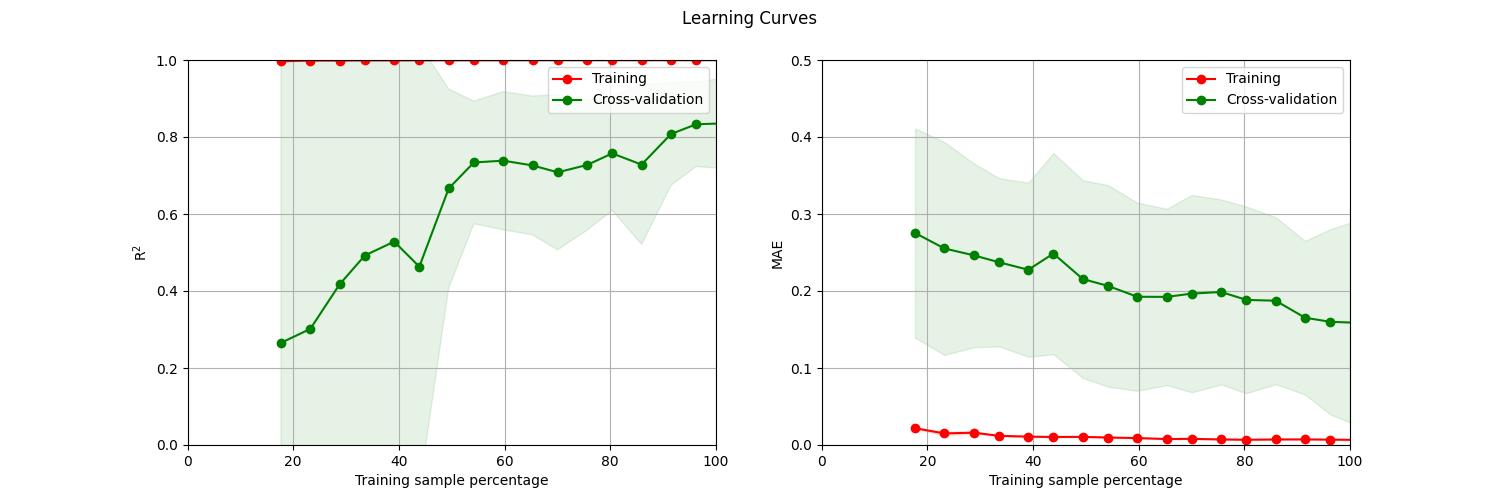 |
| **BaggingRegressor**  **set B** | 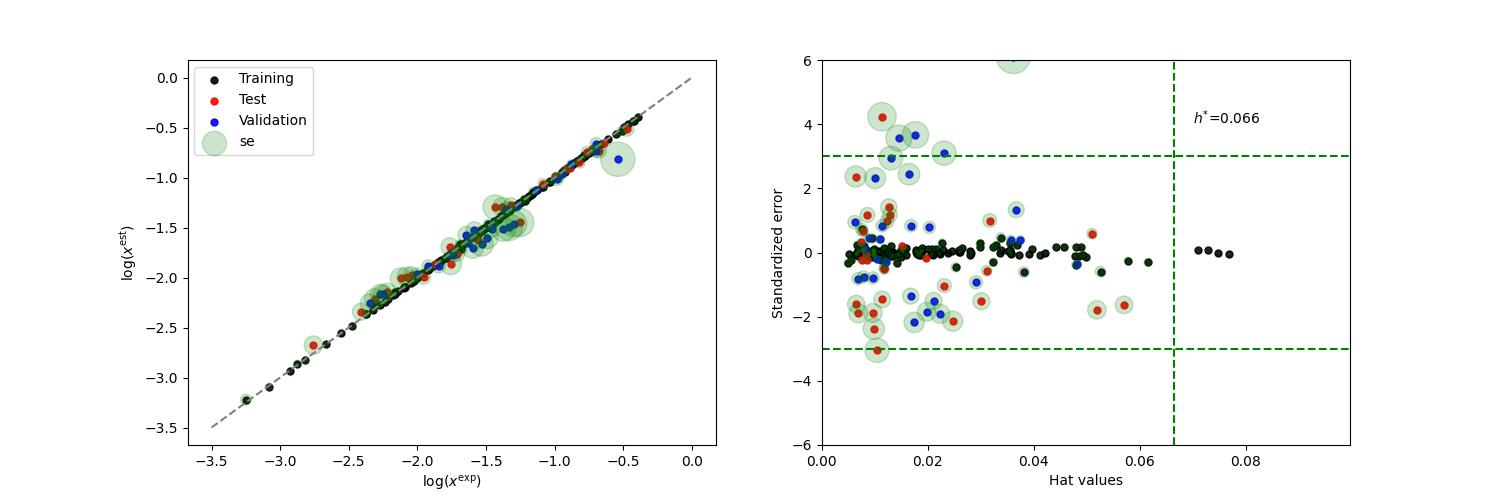  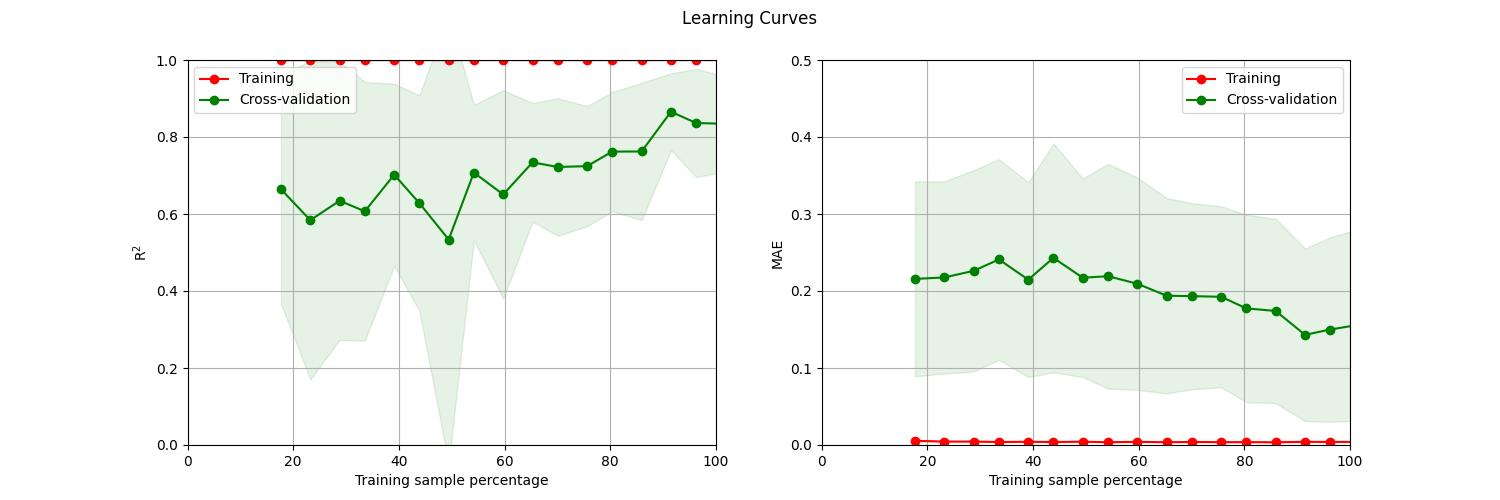 |
